# LncRNA SNHG5 can Regulate the Proliferation and Migration of Diffuse Large B Cell Lymphoma Progression via Targeting miR-181-5p/XIAP

**DOI:** 10.7150/jca.60521

**Published:** 2022-01-01

**Authors:** Xiaojing Xing, Tonghong Xu, Bin Liu, Qianxue Guo

**Affiliations:** 1 Department of Hematology and Breast Cancer, Cancer Hospital of China Medical University, Shenyang 110042, Liaoning, P.R.China.; 2 Department of Hematology and Breast Cancer, Liaoning Cancer Hospital & Institute, Shenyang 110042, Liaoning, P.R.China.

**Keywords:** SNHG5, miR-181-5p, diffuse large B cell lymphoma, competing endogenous RNA

## Abstract

**Background:** It has been verified that long noncoding RNAs (lncRNAs) may participate in the pathogenesis of various human diseases. This study aims to investigate the roles of lncRNA SNHG5 in diffuse large B cell lymphoma (DLBC), especially the impacts of lncRNA SNHG5 on proliferation and migration of human diffuse large B cell lymphoma cells and the related mechanism.

**Methods:** qRT-PCR analysis was carried out to examine the expression pattern of SNHG5 in DLBC tissue and adjacent normal tissue. The effect of SNHG5 on the proliferation, apoptosis, migration, and invasion of DLBC cells was detected by MTT, flow cytometry analysis, wound healing assay and transwell assay. The correlation between SNHG5, miR-181-5p and XIAP were certified by bioinformatics analysis, and dual-luciferase reporter assay. Furthermore, rescue assays were performed to analyze the effects of SNHG5-miR-181-5p-XIAP axis on the biological behaviors of diffuse large B cell lymphoma cells. Finally, the effects of SNHG5 axis on the growth of DLBC tumor was examined by *in vivo* analysis.

**Results:** SNHG5 was significantly upregulated in diffuse large B cell lymphoma tissues. Knockdown of SNHG5 inhibited the proliferation, migration, and invasion of diffuse large B cell lymphoma cells *in vitro* and *in vivo*. LncRNA SNHG5 acted as a ceRNA through binding with miR-181-5p in DLBC cells.

**Conclusion:** LncRNA SNHG5 may promote proliferation and migration of diffuse large B cell lymphoma cells via targeting miR-181-5p/XIAP.

## Introduction

Diffuse large B cell lymphoma (DLBC) one of the most diagnosed type of lymphoma worldwide 1-3. Like many other type of cancers, the pathogenesis of DLBC is still unclear, and there is no specific treatment method for DLBC, leading to the poor prognosis and highmortality rate of the disease 4, 5. In clinical applications, the most important reason for the high mortality rate is the metastasis as well as recurrence of DLBC that occurred in most the cases 2, 3, 6. Therefore, it is of great importance to further explore the underlying mechanism of DLBC and development new methods to improve the therapeutic efficacity as well as prognosis of the disease.

Long non-coding RNAs (LncRNAs) are a group of non-coding RNAs, and LncRNAs are often with the length of over 200 nucleotides, some of the LncRNAs can be kilobases in length 7, 8. With the in-depth investigation of the underlying mechanism LncRNAs, researchers found that LncRNAs can regulate various biological events, for example cell proliferation, migration, differentiation through affecting the gene expression at post-transcriptional level 9, 10. In the case of carcinogenesis, LncRNAs were found to be involved in the pathogenesis of many types of cancers, including DLBC 9, 11-14.

Based on the results of bioinformatic analysis using the data from TCGA database, it was found that LncRNA SNHG5 was one of the most significantly up-regulated LncRNAs in the DLBC; however, the specific underlying mechanism is still unknown. In the present study, we will explore the roles of LncRNA SNHG5 in DLBC and the possible mechanism. We hypothesized that SNHG5 may function as an onco-LncRNA in DLBC and knockdown of SNHG5 could inhibit both growth and migration of DLBC cells.

## Methods

### Patients and clinical tissue samples

A total number of 90 paired DLBC tumor samples and the adjacent normal tissues were obtained from DLBC patients at Department of Hematology and Breast Cancer, Cancer Hospital of China Medical University between Jan.2019 and Feb 2020. Serum samples of 90 healthy volunteers were also collected as the control group. The samples were stored immediately after surgery in liquid nitrogen until analyzed was performed. Patients were diagnosed with DLBC through mass biopsy or cytology, and all diagnosis meets the 2017 WHO diagnostic classification criteria for lymphoid tumors^3^. All patients are newly diagnosed, have not received relevant treatment before admission, and have no history of radiotherapy and/or chemotherapy. Exclude patients with secondary DLBC, autoimmune diseases and related histories of malignant tumors. Pathology reports of all patients were collected. The informed consent was obtained from each patient. This study was approved by the ethical committee of Cancer Hospital of China Medical University.

### Cell culture

Human normal human B-lymphocytes GM12878 and human diffuse large B cell lymphoma cell lines SU-DHL-4, SU-DHL-6 and SU-DHL-10 were purchased from American Type Culture Collection (ADLBCC, USA). The DLBC cells were cultured in the RPMI-1640 Medium (Invitrogen, Carlsbad, CA, USA). Medium was supplemented with 10% fetal bovine serum (FBS, Fisher, NY, USA) and 100 units/ml penicillin and 100 μg/ml streptomycin at 37 °C with 5% CO_2_ and 95% humidity.

### Transfection

SNHG5 over-expression plasmid was synthesized by Shanghai GenePharma Co.,Ltd (Shanghai, China). SU-DHL-4 and SU-DHL-6 cells were transfected with SNHG5 shRNA by lipofectamine 3000 (Invitrogen, Carlsbad, CA, USA) in accordance with the manufacturer's instructions. The transfection efficiency was determined by RT-qPCR method.

### RT-qPCR

The miRNeasy Mini kit (Qiagen, Valencia, USA) was used to extract total RNA from tissues and cells in accordance with the manufacturer's instructions. The concentration and quality of the RNAs were determined by NanoDrop 2000 (Thermo Fisher, Wilmington, USA). The first-strand cDNA was synthesized by TransScript first-strand cDNA synthesis supermix (Transgen, Beijing, China) in accordance with the manufacturer's instructions. RT-qPCR assay was performed using the SYBR green qPCR supermix (Applied Biosystems Life Technologies, Foster, USA) in ABI prism 7500 sequence detection system (Applied Biosystems Life Technologies, Foster, USA). Conditions of the PCR reactions were presented as below: 55 °C for 10 min, 40 cycles of 95 °C for 30 s, 55-59 °C 30 s and 72 °C for 42 s. Fold changes of the target genes was calculated by 2^-ΔΔCt^ methods (cycle threshold), and expression levels of miRNA and lncRNA/target gene were normalized by U6 and GADPH, respectively.

### Cell proliferation assay

The 5-diphenyltetrazolium bromide (MTT) assay was adopted to examine the proliferation of the DLBC cells in different groups. Briefly, cells were seeded on 96-well plates (5 × 10^3^/well) and incubated with 100 μl 0.5 mg/ml MTT for 4 h in an incubator, and precipitate was dissolved in 150 μl dimethylsulfoxide (DMSO). The optical density value of each well at 570 nm was evaluated after shaking for 10 min.

### Flow cytometry assay

At 48 h after transfection, the cells of different groups were collected and mixed with 5 μl Annexin-V-fluorescein isothiocyanate (FIDLBC) and 2.5 μl propidium iodide (PI). The apoptosis of the cells was determined by a FACSAria Sorter (Becton Dickinson, San Jose, USA).

### Scratch wound healing assay

DLBC cells were growed to 100% confluence in 6-well plates and then scratched by a 10 μl tip. After that, cells were incubated at the temperature of 37 °C with 5% CO_2_ and 95% humidified atmosphere for 24 h. The migration areas at 0 h and 24 h were imaged with an inverted microscope and analyzed by Image J.

### Transwell assay

Transwell cell invasion assay was carried out in 24-well plates using the transwell chambers (Corning Inc., Corning, USA) fitted by a 8 μm pores polyethylene terephthalate filter membrane. Cells (5 × 10^4^) were placed on the the upper chamber, and the lower chamber was filled with medium containing 10% FBS. After incubating for 24 h, cells traversed to reverse face of the membrane were stained with crystal violet and imaged. The images were captured from 5 randomly chosen fields by a microscope.

### Western blotting

The antibodies were all purchased from Santa Cruz Biotechnology (Dallas, USA). Samples (15 μg protein/lane) were electrophoresed by SDS-PAGE method and transferred onto PVDF membranes by iBlot Gel Transfer Device (Thermo Fisher). The membranes were then blocked by Blocking One (Nacalai Tesque, Kyoto, Japan) and incubated with the primary antibodies at 4 °C overnight. On day 2, the protein bands were incubated with horseradish peroxidase (HRP)-conjugated secondary antibodies (Cell Signaling Technology), and treated with ECL Prime Western Blotting Detection Reagents (GE Healthcare Life Sciences, Little Chalfont, UK). Finally, ImageQuant TL GE Healthcare Life Sciences) system was applied to digitize the strength of the band.

### Luciferase activity assay

SNHG5 cDNA and miR-181-5p fragments including microRNA binding sites was cloned into the pmirGLO plasmids (Promega, Madison, USA). Mutant SNHG5 (pmirGLO-SNHG5-MUT) and Mutant miR-181-5p (pmirGLO-miR-181-5p-MUT) were also generated as the control. Luciferase reporter plasmid and miR-181-5p mimics, XIAP mimics or miR-NC mimics were co-transfected into 293 cells by Lipofectamine 2000. 48 h after transfection, the relative luciferase activity was examined by luminometer by Dual-Luciferase Reporter Assay System (Promega).

### Xenograft nude-mice tumor models

20 Male nude mice (Balb/c-nu/nu, 4-6 weeks) were purchased from the animal center of Nanjing Medical University (Nanjing, China) and kept in specific pathogen-free (SPF) room supplied with food and water adlibitum. DLBC cells were subcutaneously injected into the right flank of the mice to create the xenograft tumor. 28 days later, the mice were sacrificed and the weight of the tumors was recorded.

### Statistical analysis

Data were expressed as mean ± SD. The comparisons between two groups or multiple groups were examined by Student's t-test or one-way analysis of variance, respectively. P < 0.05 was considered as statistical significance. The statistical analysis in this study were carried out by SPSS version 13.0 (Chicago, IL, USA).

## Results

### Upregulation of SNHG5 in DLBC serum samples and cell lines

**Figure 1:**
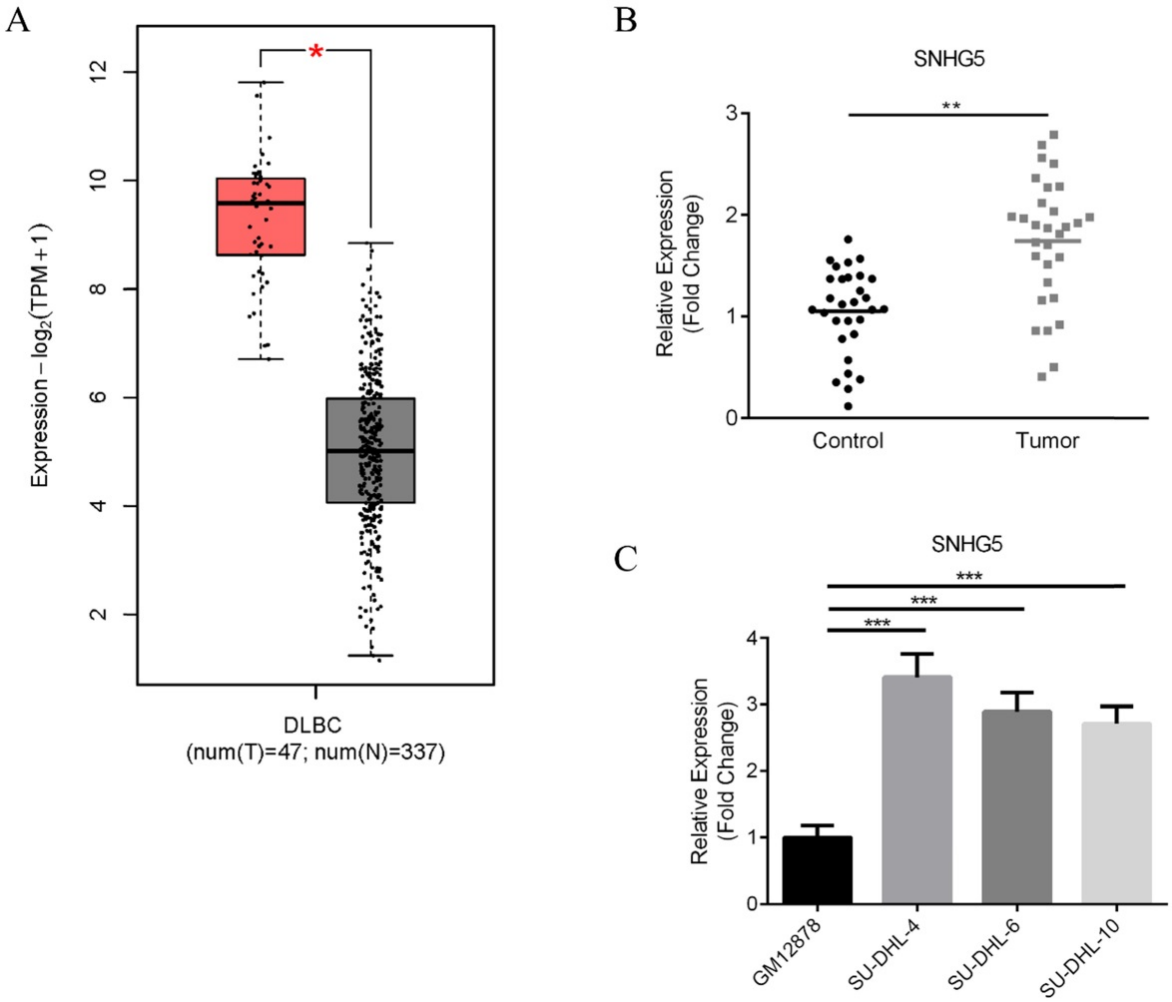
** Upregulation of SNHG5 in DLBC serum samples and cell lines.** (A) Results of bioinformatic analysis. (B) Comparison of the expression of SNHG5 in DLBC tumor samples and the adjacent normal tissues. (C) The expression of SNHG5 in DLBC cell lines and normal human B-lymphocytes. **p<0.01, ***p<0.001.

First of all, the expression of SNHG5 in DLBC tumor samples and normal tissues were compared. Figure 1 shows the results of bioinformatic analysis. We found that the expression of SNHG5 markedly increased in DLBC tumor samples compared with the normal tissue (Figure 1A, p<0.01); moreover, we collected 90 DLBC samples and adjacent normal tissues (Table 1), and the expression level of SNHG5 were compared. We found that SNHG5 were significantly increased in DLBC tumor samples compared with the normal tissue (Figure 1B, p<0.01). Furthermore, the expression of SNHG5 in DLBC cell lines and normal human B-lymphocytes were also compared. The levels of SNHG5 were increased in all DLBC cell lines (Figure 1C, p<0.05), especially SU-DHL-4, thus, it has been used for the *in vitro* studies.

### Knockdown of SNHG5 inhibits the proliferation and promotes the apoptosis of DLBC cells

**Figure 2:**
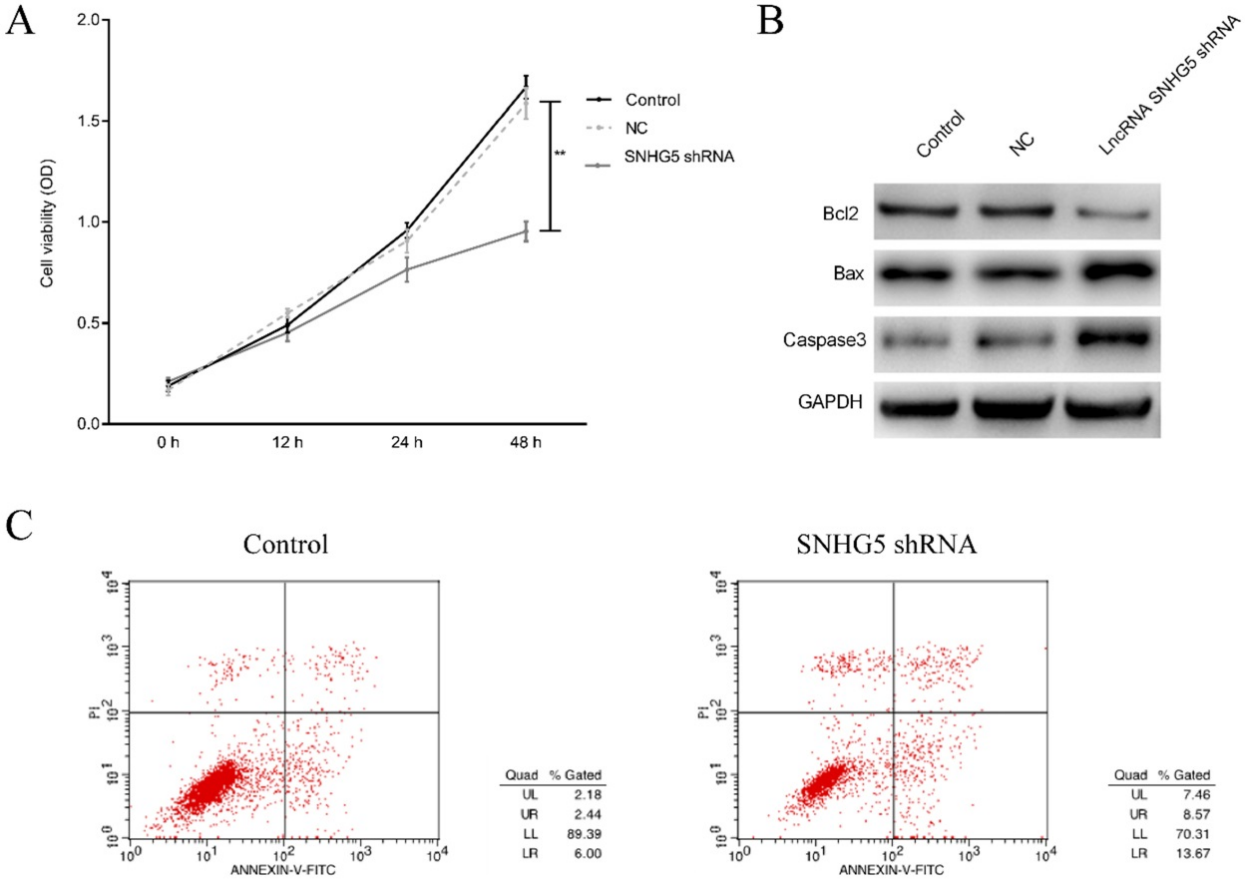
** Knockdown of SNHG5 inhibits the proliferation and promotes the apoptosis of DLBC cells.** (A) Cell proliferation by MTT assay. (B) Expressions of proliferation and apoptosis related proteins. (C)Cell apoptosis by flow cytometry assay. **p<0.01, ***p<0.001.

To investigate the roles of SNHG5 in regulating the proliferation and apoptosis of DLBC cells, DLBC cells was treated with SNHG5 shRNA, and the proliferation and apoptosis of the cells were determined. We found that SNHG5 over-expression plasmid dramatically inhibited the proliferation and promoted the apoptosis of DLBC cells *in vitro* (Figure 2A and C, p<0.01). Moreover, the expression of anti-apoptotic protein Bcl-2 was decreased and the expression of pro-apoptotic protein BAX, Caspase-3 were increased by SNHG5 shRNA (Figure 2B, p<0.01).

### Knockdown of SNHG5 inhibits the migration and invasion of DLBC cells *in vitro*

**Figure 3:**
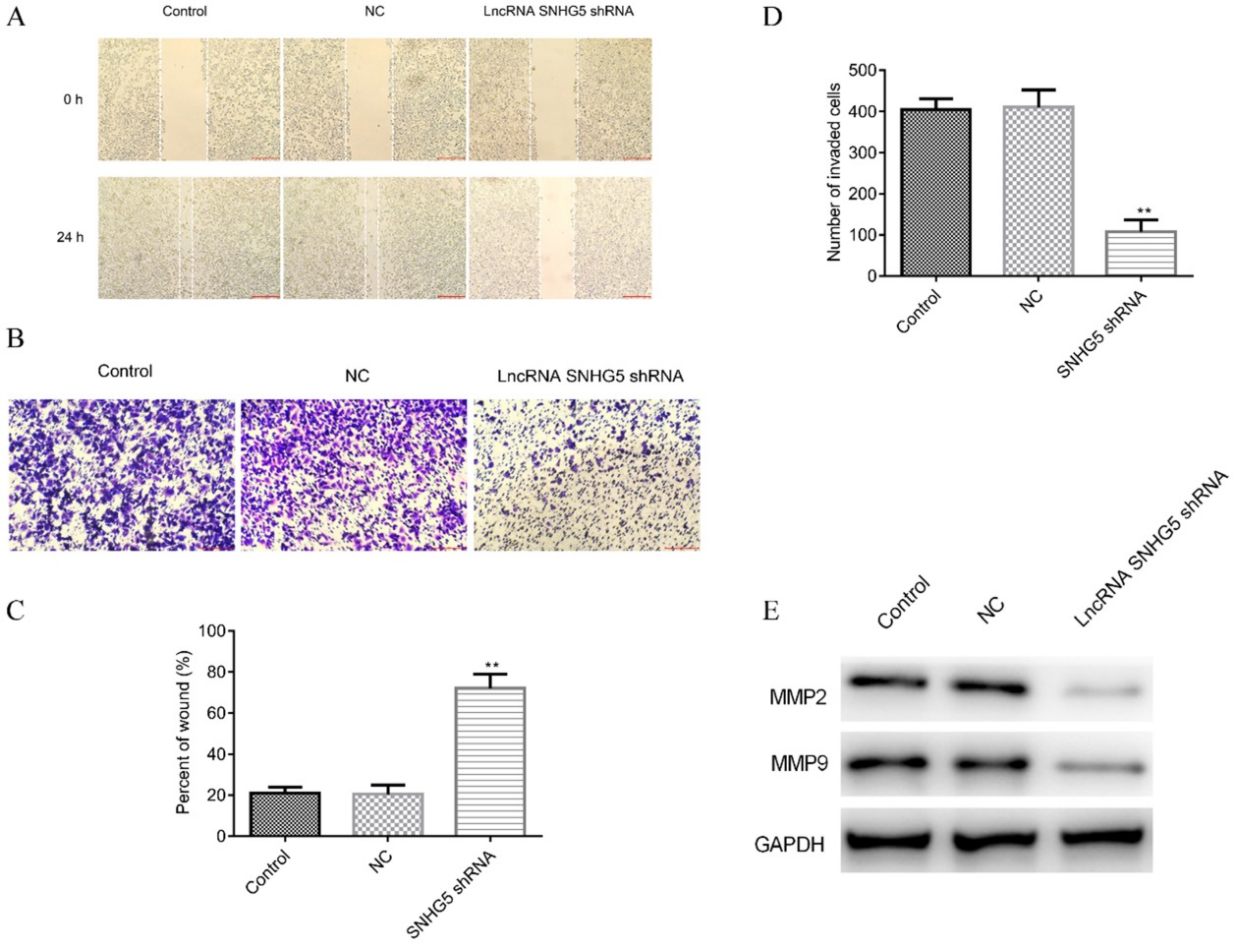
** Knockdown of SNHG5 inhibits the migration and invasion of DLBC cells *in vitro*.** (A and C) Cell migration ability by wound healing assay. (B and D) Cell invasion ability by transwell assay. (E) Expressions of migration and invasion related proteins. **p<0.01, ***p<0.001.

Next, the roles of SNHG5 in regulating the migration and invasion of DLBC cells were also explored. It has been observed that SNHG5 shRNA significantly inhibited the migration (Figure 3A and C, p<0.01) and invasion (Figure 3B and D, p<0.01) of DLBC cells *in vitro*. The expression of migration and invasion related proteins MMP-2 and MMP-9 was decreased by over-expression of SNHG5 (Figure 3E, p<0.01).

### Knockdown of SNHG5 inhibits the growth of the DLBC tumor *in vivo*

**Figure 4:**
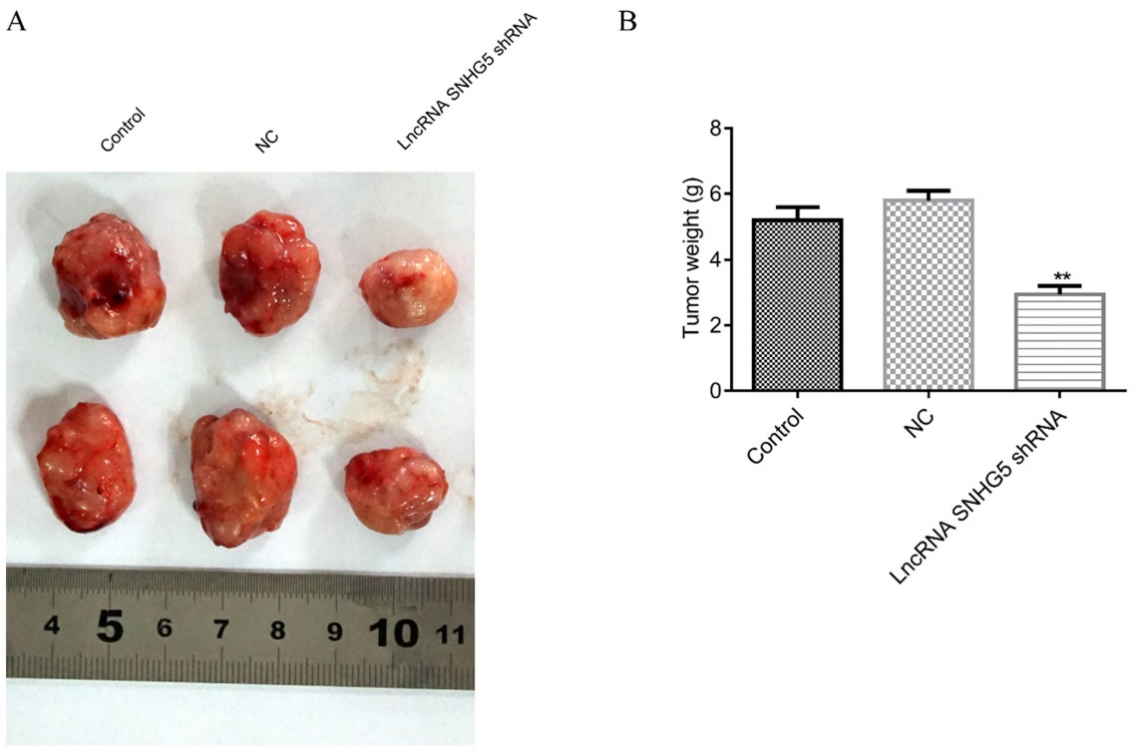
** Knockdown of SNHG5 inhibits the growth of the DLBC tumor *in vivo*.** (A and B) Weight of the tumors in different groups. **p<0.01, ***p<0.001.

Next, to the *in vivo* roles of SNHG5 in DLBC was also explored by the xenograft mice tumor models. We found that the weight of the DLBC tumors was significantly lower in SNHG5 knockdown group in comparison with the controls (Figure 4A and B, *p*<0.01), suggesting that knockdown of SNHG5 can inhibit the growth of the tumor *in vivo*.

### SNHG5 inhibits the proliferation and migration of DLBC cells via targeting miR-181-5p/XIAP

**Figure 5:**
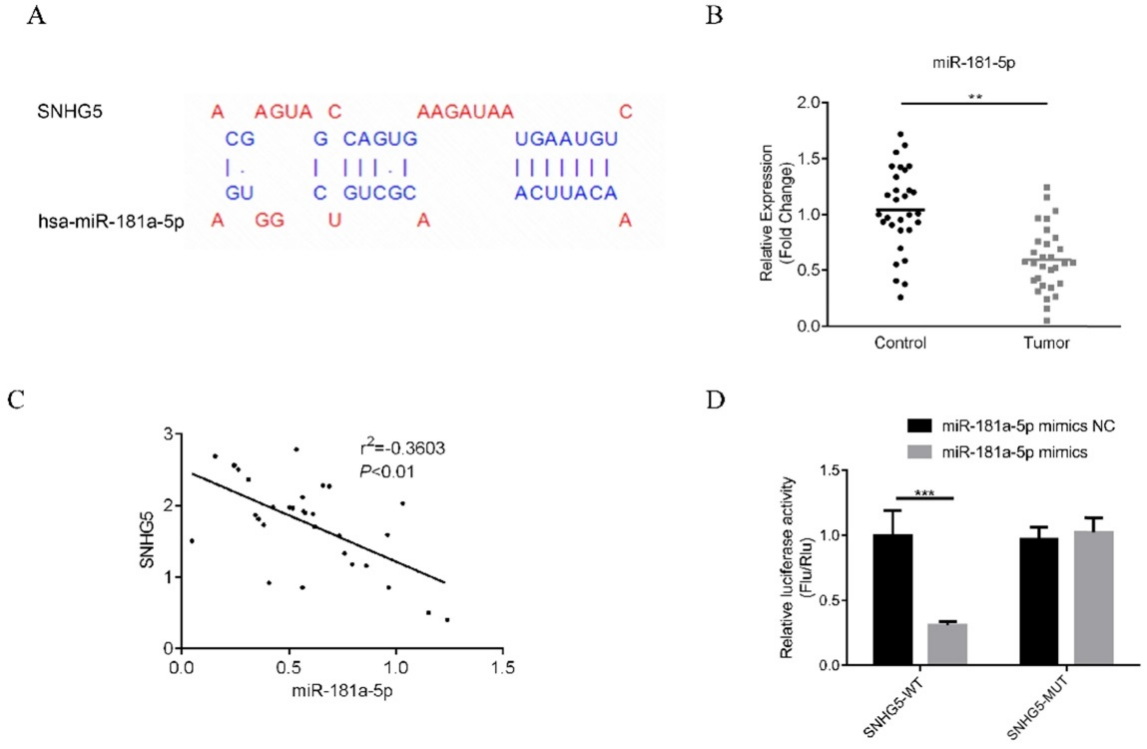
** miR-181-5p is a target of SNHG5.** (A) miR-181-5p was identified as a target of SNHG5 by bioinformatic method. (B) Expression of miR-181-5p in DLBC tissue and the adjacent normal tissue. (C) Correlation between the levels of SNHG5 and miR-181-5p in DLBC tissues. (D) Results of dual-luciferase reporter assay. **p<0.01, ***p<0.001.

**Figure 6:**
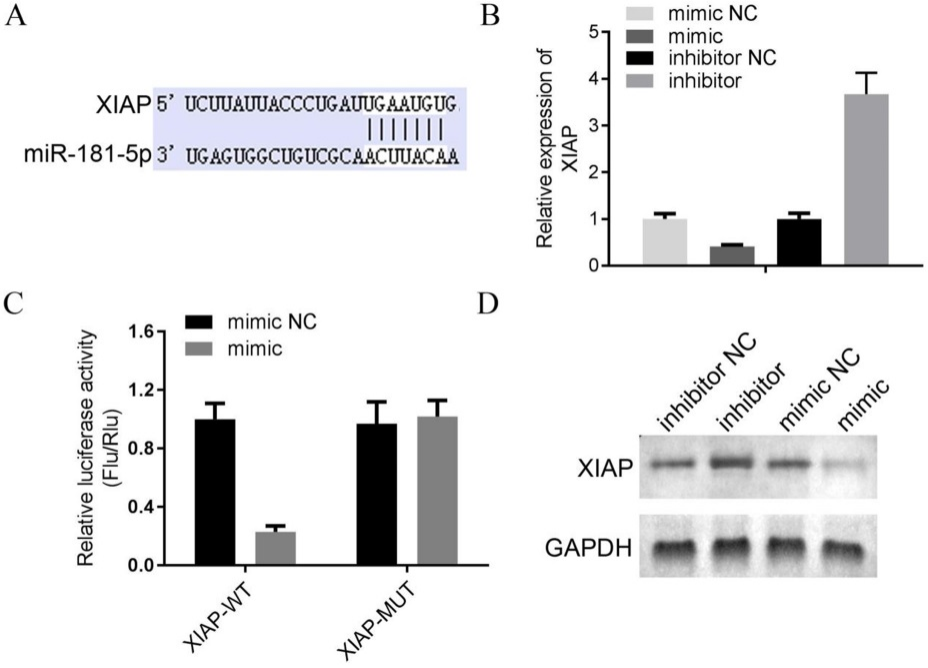
** XIAP is a target of miR-181-5p.** (A) XIAP was identified as a target of miR-181-5p by bioinformatic method. (B) The expression level of miR-181-5p was determined by qPCR in 4 DLBC lines (mimic NC, mimic, inhibitor NC and inhibitor). (C) Results of dual-luciferase reporter assay. (D) The expression level of miR-181-5p was determined by Western bolt in 4 DLBC lines (mimic NC, mimic, inhibitor NC and inhibitor).

**Figure 7:**
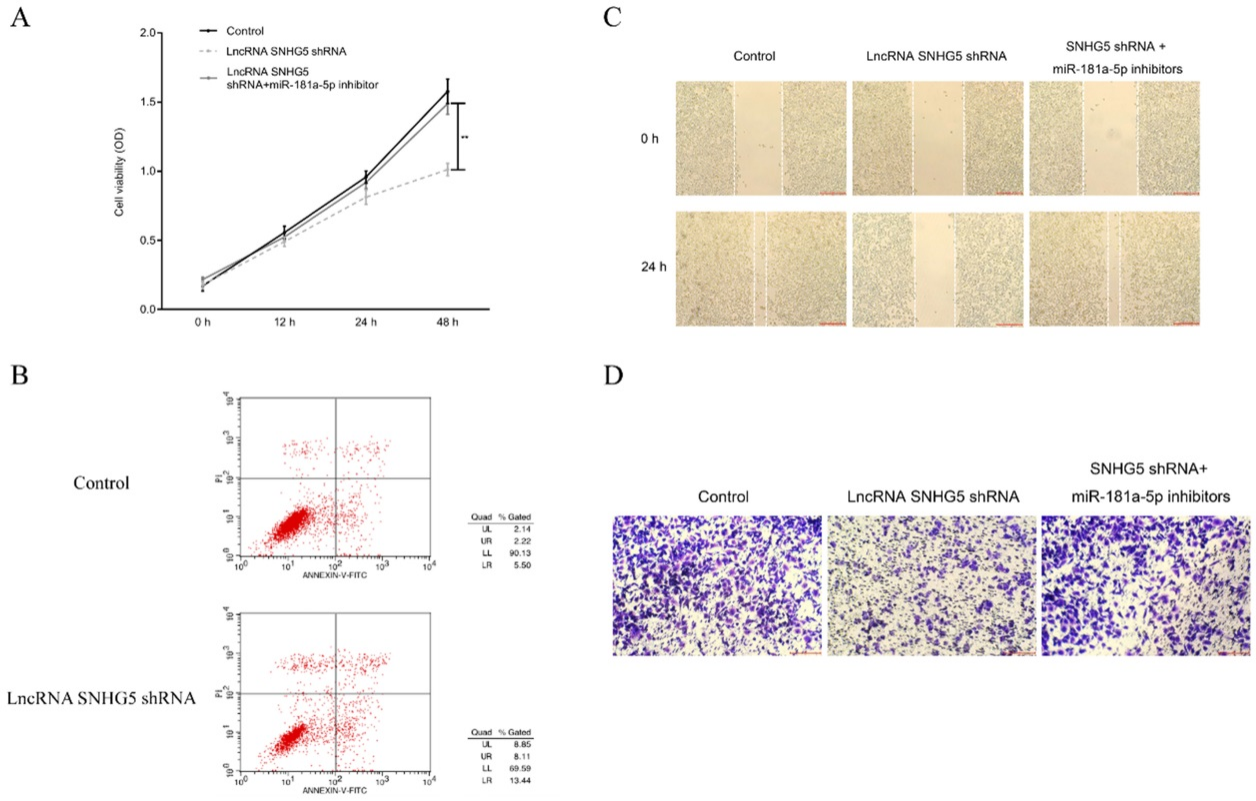
** SNHG5 inhibits the proliferation and migration of DLBC cells via targeting miR-181-5p.** (A) Cell proliferation by MTT assay. (B) Cell apoptosis by flow cytometry assay. (C) Cell migration ability by wound healing assay. (D) Cell invasion ability by transwell assay **p<0.01, ***p<0.001.

The underlying mechanism of SNHG5 as an onco-LncRNA in DLBC was explored. First, Using bioinformatics methods to predict the SNHG5/miR-181-5p/XIAP axis regulatory network in DLBC cells (Figure 5A and Figure 6A). miR-181-5p was identified as a target of SNHG5, and XIAP was identified as a target of miR-181-5p. Next, we found miR-181-5p was significantly decreased in DLBC tissue, and the expression level of SNHG5 and miR-181-5p in DLBC tissue was negatively correlated with (Figure 5B and C). Moreover, results of dual-luciferase reporter assay confirmed the targeting relationship between SNHG5 and miR-181-5p, miR-181-5p and XIAP. (Figure 5D, Figure 6B, C and D). Finally, we also found that co-transfection of SNHG5 over-expression plasmid and miR-181-5p mimics partially abrogated the anti-tumor effects of SNHG5 shRNA by promoting the proliferation (Figure 7A), migration (Figure 7C), invasion (Figure 7D), and inhibiting the apoptosis (Figure 7B) of DLBC cells.

## Discussion

In the present study, the roles of LncRNA SNHG5 in DLBC and the underlying mechanism were explored. We found that LncRNA SNHG5 may serve as an onco-LncRNA by inhibit the proliferation and migration of DLBC cells via modulating miR-181-5p/XIAP expression.

The roles of SNHG5 in different type of cancers have been discussed previously. For example, upregulation of SNHG5 may induce the angiogenesis in acute myelogenous leukemia 15; On the other hand, SNHG5 can regulate miR-205-5p expression and contributes to development of renal cell carcinoma^16^; furthermore, SNHG5 may function as an oncogenic LncRNA in nasopharyngeal carcinoma via targeting miR-1179 17. In DLBC, the roles of SNHG5 have not yet been discussed. Interestingly, results of bioinformatic analysis based on the data from the TCGA databased suggested that SNHG5 is one of the most significantly up-regulated LncRNAs in DLBC, suggesting that SNHG5 may also play carcinogenic roles in DLBC. In this study, we observed the decreased expression of SNHG5 in DLBC tumor tissue and cell lines, which was consistent with the results of the bioinformatic analysis of the TCGA databased. The above results proved that SNHG5 was over-expressed in DLBC and may sever as an onco-LncRNA.

Like any other type of cancers, the un-controlled growth and metathetic ability of DLBC lead to the high recurrent and mortality rate of the disease 18-20. Therefore, an effective molecular therapeutic target of DLBC should be a key regulator that affect the proliferation and migration of the DLBC cells. In the present study, we found that knockdown of SNHG5 significantly suppressed the carcinogenic behavior of DLBC cells by inhibiting the proliferation, migration, invasion and promoting the apoptosis of DLBC cells *in vitro* and *in vivo*. These data suggested that SNHG5 may serve as potential novel therapeutic for the targeted-therapy for DLBC.

In previous studies, the interaction between LncRNAs and miRNAs were known as the most commonly underlying mechanism for LncRNAs to exert their biological functions via “sponging” and silencing the target miRNAs 11, 21-23. With the development of the bioinformatics, the target miRNAs of LncRNAs can be predicted by virous methods. We used online bioinformatic tool starbased 3.0, and miR-181-5p has been identified as a target miRNA of SNHG5. By literate research, we found that miR-181-5p function as a tumor suppressor in different type of cancers 24-26. Therefore, we hypothesized that LncRNA SNHG5 may exert its oncogenic function via down-regulating the expression of miR-181-5p. To verify this hypothesis, we performed a series of analysis and confirmed the negative correlation relationship between the levels of miR-181-5p and SNHG5 in DLBC tumor tissue; moreover, the direct targeting relationship between SNHG5 and miR-181-5p was also confirmed by dual luciferase reporter assay. Interestingly, miR-181-5p mimics partially abrogated the anti-tumor effects of SNHG5 shRNA in DLBC cells, by promoting the proliferation, migration, invasion, and inhibiting the apoptosis of the cells *in vitro*. In order to further determine the regulatory effect of SNHG5 on DLBC cells, we analyzed the LncRNAs/miRNAs/mRNA network of SNHG5 in DLBC cells. Discover the target gene XIAP of miR-181-5p through bioinformatics analysis and dual luciferase reporter gene detection. The study by Gu et al. has shown that the abnormal expression of XIAP is related to the apoptosis of DLBC cells 27, which is similar to the regulation mechanism of SNHG5. Taken together, we speculate that there is an Lnc SNHG5/ miR-181-5p /XIAP axis in DLBC cells, and SNHG5 can serves as onco-LncRNA to regulate the development of DLBC, which in turn promote the proliferation and migration of the cancer cells.

## Conclusion

To sum up, we reported for the first time that LncRNA SNHG5 may function as a ceRNA to promote proliferation, migration, and invasion of diffuse large B cell lymphoma cells through modulating miR-181-5p expression. Our data suggested that targeting the SNHG5/miR-181-5p/XIAP axis may be a potential method for the treatment of DLBC.

## Figures and Tables

**Table 1:** Associations between the expression level of SNHG5 and clinical characteristics of DLBCL patients (n=90).

Characteristic	N (%)		P value
	Low (40)	High (50)	
Sex			0.3961
male	22	23	
female	18	27	
Age			0.4985
≤60	26	29	
60	14	21	
Ann Arbor stages			0.0339
I-II	25	20	
III-IV	15	30	
ECOG			0.4479
0-1	24	26	
≥2	16	24	
IPI score			0.0025*
0-2	28	19	
3-5	12	31	
LDH ratio			0.2076
≤1	21	32	
>1	19	18	
